# Root-growth Characteristics Contributing to Genotypic Variation in Nitrogen Efficiency of Bottle Gourd and Rootstock Potential for Watermelon

**DOI:** 10.3390/plants8030077

**Published:** 2019-03-25

**Authors:** Abdullah Ulas, Esat Doganci, Firdes Ulas, Halit Yetisir

**Affiliations:** 1 Soil Science and Plant Nutrition Department, Agricultural Faculty, Erciyes University, Kayseri 38039, Turkey; 2 Horticulture Department, Agricultural Faculty, Erciyes University, Kayseri 38039, Turkey; edoganci@gubretas.com.tr (E.D.); fulas@erciyes.edu.tr (F.U.); yetisir1@erciyes.edu.tr (H.Y.)

**Keywords:** N-efficiency, bottle gourd, grafting, rootstock, watermelon, root morphology

## Abstract

In this study, two hydroponic experiments were conducted in nutrient solution growth system. Experiments were conducted in growth chamber of Erciyes University, Agricultural Faculty in Kayseri, Turkey. In the first experiment, 10 local Turkish bottle gourd genotypes and two commercial watermelon cultivars were screened under 2 N doses (0.3 mM and 3.0 mM N) in RBD design with three replications for six weeks. In the second experiment, four genotypes (N-efficient: 70-07 and 07-45, N-inefficient: 35-10 and 45-07) were selected and used as rootstock for grafting with N-inefficient watermelon cultivar (Crimson Sweet) under 2 N doses. The grafted N-efficient gourd genotypes (07-45 and 70-07) significantly contributed to growth and biomass production of the N-inefficient watermelon plants as compared to non-grafted control plants and thus showed a higher rootstock potential for watermelon. The N-efficiency of some gourd genotypes was associated with vigor root growth and active root system particularly at low N conditions. These traits could be useful characters to select ‘N-efficient’ bottle gourd rootstocks for sustainable agriculture in the future.

## 1. Introduction

Sustainability in food and agricultural production depends on the efficient use of natural resources. However, to keep food production at the same level as population growth without using up or devastating the natural resources is not an easy task for sustainable agriculture. Moreover, world population is increasing at a rate of 77 million per year and expected to be around 8.6 billion for the year 2030 and 9.6 billion for 2050 [1]. To meet the growing food demand of the population, food production has to increase over 70% from the current level [2]. An increase in food production requires higher fertilizer application. Particularly, nitrogen (N) is the quantitatively most important nutrient [3] which has an immediate effect on crop growth and yield improvement. Therefore, to secure yields, growers apply more fertilizer than recommended [4] and so the global use of chemical N fertilizers in the world increased largely during the past four decades [2]. On the other hand, the available N is often limiting plant growth more than any other nutrient in both high-input and low-input agriculture systems [5].

Due to insufficient fertilizer application by small-scale farmers, soil fertility is declining in some regions. On the other hand, environmental pollution of both air and water due to use of intensive N fertilizers in high-input agriculture has to be considered [6]. However, the efficiency of N fertilizers is frequently low, since, plants take up often less than 50% of the applied N [7], and the proportion of fertilizer N not utilized by the crop is left in the soil and/or lost from the plant/soil system through volatilisation, leaching and denitrification [2,8]. Therefore, to ensure improved utilization of available N in sustainable agriculture, improved fertilizers as well as soil and crop management practices are necessary [9]. Among these, a way to improve N efficiency and reduce the losses in production caused by low N efficiency in high-yielding genotypes would be to graft them onto rootstocks capable of improving N efficiency of the scion in both low-input and high-input agriculture systems.

The nutrient efficiency is related to the genetic variation among the crop plants and has been well known for at least 92 years [10]. Recently, due to ecological, economical and socio-economical grounds, many plant physiologists and plant breeders draw increasing attention to genotypic differences in nutrient efficiency. Nutrient efficiency is an ill-defined expression in literature. In this present work, “N-efficient” genotypes were defined as those realizing an above-average yield under suboptimal N supply [11], while genotypes having a high yield under optimum N supply were called “N-responsive” [12]. The aim of this study was to determine the genotypic differences in N efficiency of some local bottle gourd genotypes (*Expt. 1.*) and to evaluate whether grafting could improve N efficiency of watermelon (*Expt. 2.*) through examining the changes induced by the rootstock in the shoot growth at agronomical, root growth at morphological and leaf development at physiological levels.

## 2. Materials and Methods

### 2.1. Plant Material, Treatments and Experimental Design

Two hydroponic experiments (*Exp. 1* and *Exp. 2*) were conducted by using a non-flow nutrient film technique (NFT) in a controlled growth chamber situated in the Plant Physiology Laboratory of Erciyes University, Faculty of Agriculture, central Anatolia in Turkey. In *Exp. 1* (April 2016), 10 Turkish bottle gourd (*Lagenaria siceraria*) germplasm genotypes which are characterized as salt-tolerant (07-45, 42-11, 45-04, 47-04,70-07) and salt-sensitive (01-18, 35-10, 41-01, 45-07, TR-50), were tested together with two commercial watermelon cultivars (Crimson Sweet and Sugar Baby). With regards to results of *Exp. 1*, four gourd genotypes (N-efficient: 70-07 and 07-45, N-inefficient: 35-10 and 45-07) were selected as rootstocks while the watermelon cultivar Crimson Sweet (N-inefficient) was used as scion in the second experiment (*Exp. 2*) conducted for grafting. To produce homogenous plantlets for hydroponic growth medium, seeds of watermelon were sown one week earlier than quickly germinating bottle gourd’s seeds in a multi-pot contained a mixture of peat (pH: 6.0-6.5) and perlite in a 2:1 ratio for two weeks. The seedlings with two-true-leaves were carefully freed from the peat-perlite growth medium with no root damage and then transferred into 8 L plastic pots filled with nutrient solution in growth chamber. In both hydroponic experiments the total vegetation period from transplanting into 8 L plastic pots up to final harvest was almost six weeks. The average day/night temperatures were 25/22 °C, the relative humidity was 65–70% and about 350 µmol m^−2^ S^−1^ photon flux was supplied in a photoperiod of 16/8 h of light/dark regimes in the controlled growth chamber. Two hydroponic experiments were arranged in a completely randomized block design with four replications and three plants in each pot (replication). In both experiments, all nutrients were supplied to the plants by using a non-flow nutrient film technique (NFT) applied in 8 L plastic pots. The nutrient solution was prepared by using distilled water containing analytical grade (99% pure) chemicals according to a modified Hoagland and Arnon formulation. Nutrient solution in each 8 L pot was continuously aerated by using an air pump. In both hydroponic experiments, nitrogen was supplied in two different concentrations (Low N: 0.3 mM N, High N: 3.0 mM N) by using two different proportional N sources (75% Ca(NO_3_)_2_ and 25% (NH_4_)_2_SO_4_). Furthermore, basic nutrient solution had the following composition (μM): K_2_SO_4_ (500); KH_2_PO_4_ (250); CaSO_4_ (1000); MgSO_4_ (325); NaCl (50); H_3_BO_3_ (8); MnSO_4_ (0.4); ZnSO_4_ (0.4); CuSO_4_ (0.4); MoNa_2_O_4_ (0.4); Fe-EDDHA (80). All nutrients were replaced when the N concentration of the nutrient solution in the 3.0 mM N rate pots fell below 0.3 mM, as measured daily with nitrate test strips (Merck, Darmstadt, Germany) by using a NitracheckTM reflectometer.

### 2.2. Harvest, Shoot and Root Dry Weight Measurments

In both hydroponic experiments (*Exp. 1* and *Exp. 2*) three plants per pot were harvested 42 days after treatment (DAT). Harvested plants were separated into shoot and roots. For the fresh and dry weight determination, the shoot was fractioned into the leaf, stem and roots. Plant materials were dried in a forced-air oven for 48 h at 70 °C to determine dry weights.

### 2.3. Root Morphological Measurements

In both hydroponic experiments, the plant root morphological parameters such as root length (m) and root volume (cm^3^) of the plants were measured by using a special image analysis software program WinRHIZO (Win/Mac RHIZO Pro V. 2002c Regent Instruments Inc., Québec, QC G1V 1V4, Canada) in combination with Epson Expression 11000XL scanner. From each harvested fresh root samples almost 5.0 g sub-samples were taken. The samples were each (one after the other) placed in the scanner’s tray. Water was added and with the aid of a plastic forceps, the roots were homogenously spread across the tray; and the scanning and analysis done from the WinRhizo system’s interface on a computer connected to the scanner. The total plant root length and volume was then determined as the ratio of sampled root fresh weight to the total root fresh weight.

### 2.4. Leaf Area and Photosynthetic Activity Measurements

In both hydroponic experiments, leaf area of the plants was measured destructively during the harvesting process by using a portable leaf-area meter (LI-3100, LI-COR. Inc., Lincoln, NE, USA). Total leaf area was recorded in centimeter square (cm^2^) and converted into meter square (m^2^). Prior to harvest, non-destructive measurements of the leaf-level CO_2_ gas exchange (µmol CO_2_ m^−2^ s^−1^) were done in controlled growth chamber by using a portable photosynthesis system (LI-6400XT; LI-COR Inc., Lincoln, NE, USA). The leaf net photosynthesis measurement was performed on the youngest fully expanded leaves, using four replicate leaves per treatment in third and fifth week of the vegetation period.

### 2.5. Shoot Nitrate Reductase (NRA) Activity Measurement

Nitrate reductase (NRA) activity in the shoot was determined following the method proposed by Harley [13]. At harvesting fresh plant samples were taken and chopped into pieces; two grams of the latter were placed in each of two falcon tubes and labeled time-0 (T0) and time-60 (T60). The tubes were covered with aluminum foil to be screened from light. Ten ml of assay buffer solution [100 mM phosphate buffer, pH 7.5; 30 mM KNO_3_; 5%(*v/v*) propanol] was added to each tube (T0 and T60). The T0 container was immediately placed into boiling water for five minutes, removed and allowed to cool to room temperature. While the T60 was kept for 60 minutes at room temperature; after which it was also placed into boiling water for five minutes and allowed to cool to room temperature. To detect nitrite in the assay tubes, the optical density (OD) of each standard tube was determined at 540 nm wavelength in the spectrometer.

### 2.6. Shoot Nitrogen Analysis

After grinding shoot dry materials, almost 200 mg from each dry plant samples were taken to analyze the shoot N concentration (mg N g^−1^d.w.) by using Kjeldahl Nitrogen Determination Method, introduced by Johan Kjeldahl in 1883 [14]. After determination of shoot N concentration, the value was multiplied by total shoot dry matter in order to calculate the total shoot N content (N uptake) of a whole plant (mg N plant^−1^).

### 2.7. Statistical Analysis

Statistical analysis of the both nutrient solution experiments data was performed using SAS Statistical Software (SAS 9.0, SAS Institute Inc., Cary, NC, USA). A two-factorial analysis of variance was performed to study the effects of genotype (*Exp. 1*) or grafting combination (*Exp. 2*) and nitrogen and their interactions on the variables analyzed. Levels of significance are represented by * *p* < 0.05, ** *p* < 0.01, *** *p* < 0.001, and ns means not significant. Differences between the treatments were analyzed using Duncan’s Multiple Test (*p* < 0.05).

## 3. Results and Discussion

### 3.1. Screening Experiment (Exp. 1)

**Figure 1:**
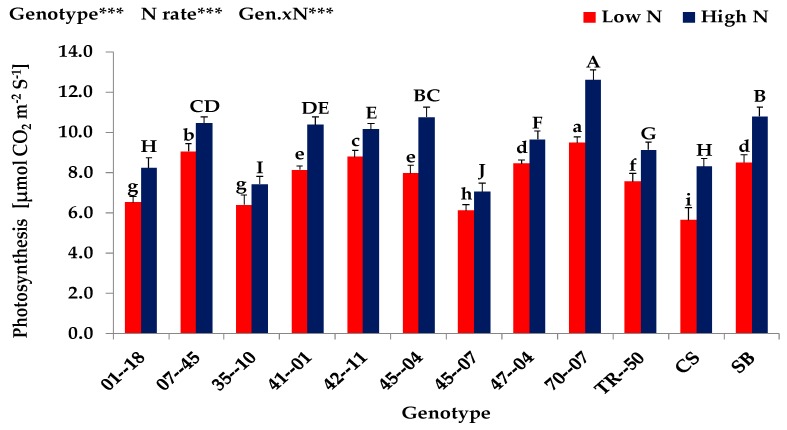
Photosynthesis of the ten bottle gourd genotypes and two watermelon cultivars grown under low (0.3 mM) and high (3.0 mM) N supply in *Expt. 1*. Values denoted by different letters (lower- and upper-case letters for low and high N supply, respectively) are significantly different between genotypes within columns at *p* < 0.05. ns, non-significant. * *p* < 0.05, ** *p* < 0.01 and *** *p* < 0.001.

**Figure 2:**
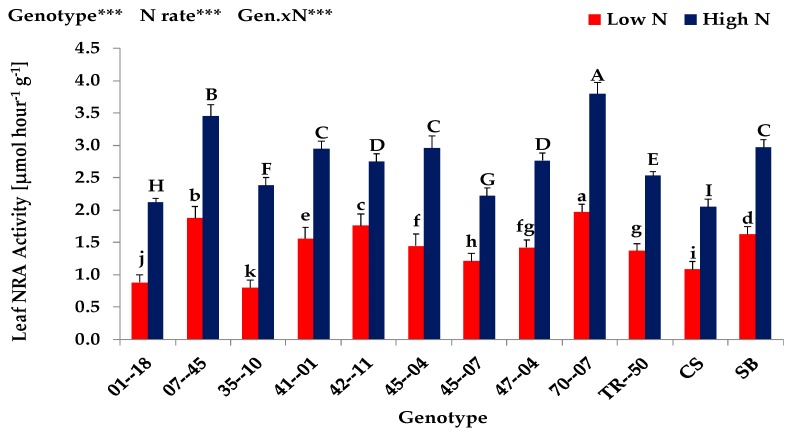
Leaf NRA activity of the ten bottle gourd genotypes and two watermelon cultivars grown under low (0.3 mM) and high (3.0 mM) N supply in *Expt. 1*. Values denoted by different letters (lower- and upper-case letters for low and high N supply, respectively) are significantly different between genotypes within columns at *p* < 0.05. ns, non-significant. * *p* < 0.05, ** *p* < 0.01 and *** *p* < 0.001.

The results obtained from the *Expt. 1* indicated that shoot dry matter, shoot N uptake, shoot N concentration, total leaf area, photosynthesis and leaf nitrate reductase (NRA) activity of 10 gourd genotypes and two watermelon cultivars were significantly (*p* < 0.001) affected by different rates of N supply (Table 1, Figure 1 and Figure 2). Plants under high N supply showed usually a higher performance in shoot growth, N uptake, leaf area development, photosynthesis and enzyme activity than plants grown under low N condition. Increasing N supply from low to high level, led to an increase in shoot dry matter by almost 261%, in shoot N uptake by almost 527%, in shoot N concentration by almost 73%, in total leaf area by almost 359%, in photosynthesis by almost 24% and in leaf NRA activity by almost 109%. In agreement with several studies [15,16,17,18], our results clearly indicated that nitrogen has a pronounced positive effect on shoot growth and shoot N uptake which might have contributed to a high leaf area formation and thus a high photosynthetic and enzymatic (NRA) activity of leaves. Furthermore, the N availability during growth and development plays a major role in establishing and maintaining a photosynthetic active canopy [19].

In general, an optimal external N supply has a substantial effect on the leaf area [20] and leaf area duration that play an important role for the light interception and carbon assimilation by crops [21]. Consequently, biomass production and yield of a crop is strongly dependent on its leaf area as well as the rate of leaf photosynthesis [22]. On the other hand, a suboptimal supply of N usually limits the leaf growth rate and thus the leaf area index (LAI) due to low rates of net photosynthesis, insufficient cell expansion, or both of these factors [23]. In agreement with all these studies, our results clearly indicated that an increase in N supply has an encouraging contribution to shoot dry biomass, shoot N uptake, shoot N concentration, total leaf area, photosynthesis and NRA activity.

The variations among genotypes and the growth response to supplied N, i.e., the interaction between N supply and genotypes were also highly significant (*p* < 0.001) in shoot dry matter, shoot N uptake, shoot N concentration, total leaf area, photosynthesis and leaf nitrate reductase (NRA) activity (Table 1, Figure 1 and Figure 2) The variation among genotypes was usually significant, but smaller at low than high N condition. The exhibited variation ranged between 8.9 and 35.1 g/plant in shoot dry matter, 126.7 and 1003.6 mg/plant in shoot N uptake, 6.8 and 9.4 mg/g in shoot N concentration, 0.11 and 1.14 m^2^/plant in total leaf area, 3.84 and 5.56 µmol/m^2^/s in photosynthesis and 1.20 and 1.73 µmol/hour/g in NRA activity at low and high N rates, respectively. The highest shoot dry matter was shown by gourd genotypes 70-07 and 07-45 while the lowest was shown by 35-10 and 45-07 at low N supply. These results clearly indicate that both gourd genotypes 70-07 and 07-4 can be characterized as ‘N-efficient’ due to realizing an above-average yield (shoot dry biomass) under suboptimal (low) N supply [11]. Based on this reference, the lowest shoot dry biomass yielding gourd genotypes 35-10 and 45-07 can be characterized as ‘N-inefficient’.

As well, the response to supplied high N was highly significant (*p* < 0.001) among genotypes in shoot dry matter production (Table 1). At this N rate, the highest shoot dry matter was shown by three gourd genotypes 70-07, 07-45 and 42-11 while the lowest was shown again by 35-10 and 45-07 at high N supply. Based on these results, three gourd genotypes 70-07, 07-45 and 42-11 can be characterized as ‘N-responsive’ due to realizing an above-average yield (shoot dry biomass) under high N supply [12]. On the other hand, the low shoot biomass yielding gourd genotypes (35-10 and 45-07) can be characterized as ‘N-irresponsive’.

Similar to several previous field and nutrient solution studies using different field crops, wide genotypic differences in N efficiency were found in this study. For instance, significant differences in grain yield among 12 old and new wheat genotypes were reported by [24] for low and high N conditions. A comparison of 25 maize hybrids indicated significant yield variations among hybrids at low and high N supply, with highly significant genotype x N interaction for grain yield as well as for N uptake and N-utilization [25]. The study by [26] elucidated significant genotypic differences in grain yield and total N uptake of 26 Ethiopian barley genotypes under two N rates. The field and nutrient solution studies of [27] demonstrated significant genetic diversity among five sorghum cultivars in grain yield that correlated mostly with leaf canopy structure and leaf N concentrations at low N supply. Significant genotypic variations in shoot and root growth among two contrasting potato cultivars (‘Astrid’ N-efficient, ‘Bodenkraft’ N-inefficient) grown in nutrient solution at three (0.05, 0.5 ve 5.0 mM) N rates were reported by [12].

Similar to shoot dry biomass production, the ‘N-efficient’ gourd genotypes 70-07 and 07-45 showed the significantly highest shoot N uptake, total leaf area, photosynthesis and leaf NRA activity while the lowest was shown by the ‘N-inefficient’ genotypes 35-10 and 45-07 at low N condition.

Interestingly, the low yielding ‘N-inefficient’ genotypes showed substantially higher shoot N concentrations than the high yielding ‘N-efficient’ genotypes (Table 1). This might be due to a high leaf area formation which usually leads to a reduction in the amount of N per unit of leaf area [23]. This was confirmed by the results of two watermelon cultivars (cv.) that exhibited the significantly lowest shoot dry biomass yield, shoot N uptake, total leaf area, photosynthesis and leaf NRA activity, whereas surprisingly the highest shoot N concentration compared to all of 10 gourd genotypes at low and high N rates. Furthermore, two watermelon cultivars differed significantly and hence the cv. Sugar Baby produced significantly higher shoot dry matter, shoot N uptake, shoot N concentration, total leaf area, photosynthesis and leaf NRA activity than cv. Crimson Sweet at both low and high N rates. 

The results obtained from the *Expt. 1* indicated that opposite to shoot growth, the root dry matter, root:shoot ratio, total root length, total root volume of 10 gourd genotypes and two watermelon cultivars were significantly (*p* < 0.001) but negatively affected by different rates of N supply (Table 2). Plants under high N supply showed usually a lower performance in root growth and root morphological development than plants grown under low N condition. Increasing N supply, reversely led to a decline in total root dry matter by almost 20.3%, the root:shoot ratio by almost 78.4%, the total root length by almost 21.0% and the total root volume by almost 29.2% at high N condition. This might be due to a higher partitioning of dry matter to the root system under low N condition [12,15,28]. Furthermore, an increased carbohydrate sink strength of the roots under N deficiency usually leads to a greater allocation of photoassimilates to the roots [29]. Moreover, nitrogen uptake is regulated by the demand of the growing crop if N supply is not limited [30], but by the extent and effectiveness of the root system [31] and also morphological root characteristics such as maximum rooting depth, and root length density at deeper soil layers [12,32], if N is limiting. Our results corroborate those studies.

The variations among genotypes and the growth response to supplied N, i.e., the interaction between N supply and genotypes, were also highly significant (*p* < 0.001) for root dry matter, root:shoot ratio, total root length and total root volume (Table 2). Opposite to variation in shoot growth, the variation in root growth and morphology among genotypes was usually significant but relatively small at high compared to low N conditions. This is in agreement with the study of [31] who reported high root effectiveness under limiting N condition. 

The exhibited variation ranged between 6.6 and 5.4 g/plant in root dry matter, 0.46 and 0.08 g/g in root:shoot ratio, 500.6 and 436.1 m/plant in total root length and 117.3 and 88.0 cm^3^/plant in total root volume at low and high N rates, respectively. The highest root dry matter, total root length and total root volume was shown by the ‘N efficient’ characterized gourd genotypes 70-07 and 07-45 while the lowest was shown by the ‘N-inefficient’ characterized gourd genotypes 35-10 and 45-07 at low N supply. Similar results were exhibited under high N supply and thus ‘N-responsive’ characterized gourd genotypes (70-07 and 07-45) showed higher performance in root growth and morphology than ‘N-irresponsive’ characterized gourd genotypes (35-10 and 45-07). Furthermore, two watermelon cultivars (cv.) showed significantly lowest root dry biomass yield, root:shoot ratio, total root length and total root volume as compared to all of 10 gourd genotypes at low and high N rates. Also, two cultivars differed significantly and thus the cv. Sugar Baby showed significantly higher root growth and morphology than cv. Crimson Sweet at both low and high N rates. All these results suggested for us to develop a theory which proposes that a low yielding ‘N-inefficient’ watermelon cultivar (Crimson Sweet) can be improved when it is grafted onto ‘N-efficient’ gourd rootstocks (70-07 and 07-45). Based on this hypothesis, a second experiment (*Exp. 2*) was conducted by grafting an ‘N-inefficient’ watermelon cultivar (C. Sweet) onto four different gourd rootstock genotypes (70-07, 07-45, 35-10, 45-07) in this present study (see Materials and Methods).

### 3.2. Grafting Experiment (Exp. 2)

**Figure 3:**
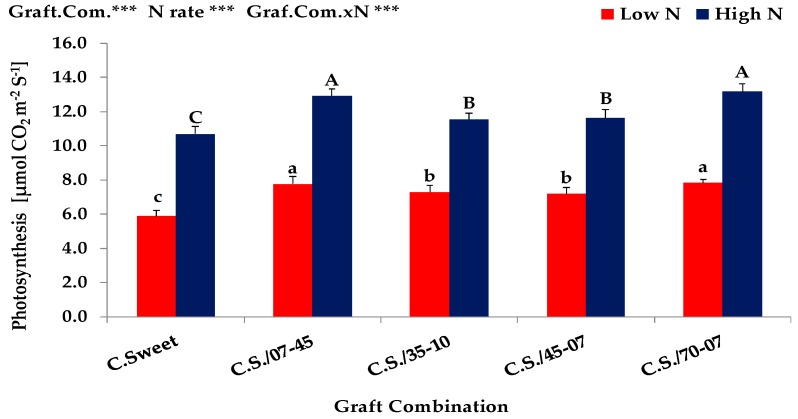
Photosynthesis of non-grafted and grafted watermelon cultivar (Crimson Sweet) onto four bottle gourd genotypes grown under low (0.3 mM) and high (3.0 mM) N supply in *Expt. 2*. Values denoted by different letters (lower- and upper-case letters for low and high N supply, respectively) are significantly different between genotypes within columns at *p* < 0.05. ns, non-significant. * *p* < 0.05, ** *p* < 0.01 and *** *p* < 0.001.

**Figure 4:**
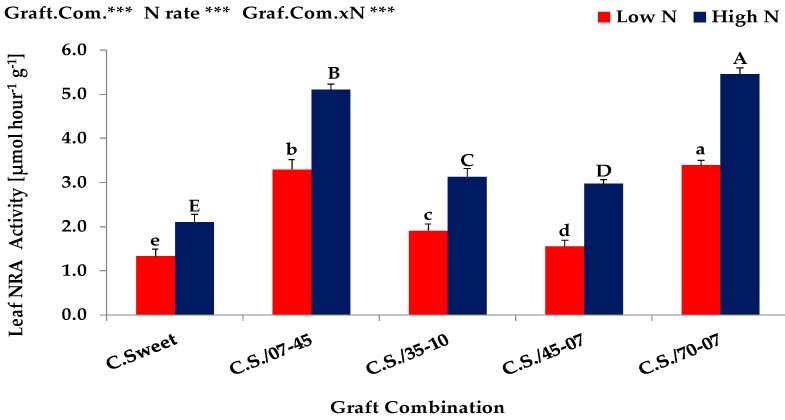
Leaf NRA activity of non-grafted and grafted watermelon cultivar (Crimson Sweet) onto four bottle gourd genotypes grown under low (0.3 mM) and high (3.0 mM) N supply in *Expt. 2*. Values denoted by different letters (lower- and upper-case letters for low and high N supply, respectively) are significantly different between genotypes within columns at *p* < 0.05. ns, non-significant. * *p* < 0.05, **P < 0.01 and *** *p* < 0.001.

The results obtained from the *Expt. 2* indicated that shoot dry matter, shoot N uptake, shoot N concentration, total leaf area, photosynthesis and leaf nitrate reductase (NRA) activity of graft combinations were significantly (*p* < 0.001) affected by different rates of N supply (Table 3, Figure 3 and Figure 4). Irrespective of graft combinations, plants under high N supply exhibited usually an improved performance in shoot growth, N uptake, leaf area development, photosynthesis and leaf NRA activity than plants grown under low N condition. Increasing N supply increased the shoot dry matter by almost 166%, the shoot N uptake 414%, the shoot N concentration 91%, the total leaf area 183%, the leaf photosynthesis 67% and the NRA activity 63% at high N condition. In agreement with several studies [15,16,17,18], our results clearly indicated that nitrogen has a pronounced positive effect on shoot growth and shoot N uptake which might have contributed to a high leaf area formation and thus a high photosynthetic and enzymatic (NRA) activity of leaves. Since, the N availability during growth and development plays a major role in establishing and maintaining a photosynthetic active canopy [19].

Similar to nitrogen impact, grafting also had significantly positive effects and therefore the grafted plants showed significantly higher performance in shoot growth, N uptake, leaf area development, photosynthesis and leaf NRA activity than non-grafted control plants grown at both low and high N rates (Table 3, Figure 3 and Figure 4). As compared to non-grafted control plants, the grafting increased (maximum) the shoot dry matter by almost 146% and 293%, the shoot N uptake by 146% and 282%, the total leaf area by 78% and 336%, leaf photosynthesis by 33% and 23% and the NRA activity by 156% and 160% at low and high N rate, respectively. These results highly corroborate several grafting experiments [33,34], conducted to increase the growth and the tolerance of various scions (melon and water melon) to low N stress conditions.

Significant differences were found among graft combinations and the growth response to supplied N, i.e., the interaction between N supply and graft combination was also highly significant (*p* < 0.001) (Table 3, Figure 3 and Figure 4). Compared to non-grafted control plant (Crimson Sweet), the graft combination 70-07/C.S. and 07-45/C.S. showed the highest shoot dry matter production at both low and high N rates. On the other hand, the graft combinations 35-10/C.S. and 45-07/C.S. exhibited slightly higher shoot dry matter than non-grafted control plants, however, but the production was significantly lower than the graft combinations of 70-07/C.S. and 07-45/C.S. at both low and high N conditions. All these results clearly confirmed our hypothesis which proposes that a low yielding ‘N-inefficient’ watermelon cultivar (Crimson Sweet) can be improved when it is grafted onto ‘N-efficient’ bottle gourd rootstocks (70-07 and 07-45). Furthermore, these results also confirmed that the selection and characterization of bottle gourd genotypes (N-efficient/N-responsive: 70-07 and 07-45, N-inefficient/N-irresponsive: 35-10 and 45-07) based on the results of *Exp. 1*, have been done correctly. Similar and corroborative results have been also reported by [33,34]. Similar to shoot dry biomass production, the graft combination 70-07/C.S. and 07-45/C.S. showed significantly higher shoot N uptake, total leaf area, photosynthesis and leaf NRA activity than the graft combinations of 35-10/C.S. and 45-07/C.S. at both low and high N conditions. Interestingly, the low yielding ‘N-inefficient’ genotypes showed substantially higher shoot N concentrations than high yielding ‘N-efficient’ genotypes. This might be due to high leaf area development which usually causes a reduction in the amount of N per unit of leaf area [23].

The results obtained from *Expt. 2.* indicated that opposite to shoot growth, the root dry matter, root:shoot ratio, total root length and total root volume of graft combinations were significantly (*p* < 0.001) but negatively affected by different rates of N supply (Table 4). Irrespective of graft combinations, plants under high N supply exhibited usually a lower root growth and root morphological development than plants grown under low N conditions. Increasing N supply, reversely led to a decline in total root dry matter by almost 20.8%, the root:shoot ratio by almost 67.9%, the total root length by almost 15.2% and the total root volume by almost 21.7% at high N conditions. This might be due to higher partitioning of dry matter to the root system under low N condition [12,15,28,29] and an increased carbohydrate sink strength of the roots [29]. However, opposite to nitrogen effects, grafting had significantly positive effects on root morphology and therefore increased (maximum) the root dry matter by almost 275% and 255%, root:shoot ratio 50% and 57%, the total root length 65% and 58% and root volume 183% and 211% at low and high N rates, respectively, as compared to non-grafted control plants. All these clearly showed that the contribution of grafting to scion is related to the root power of the rootstocks. Similar results have been clearly shown in the study of [33,34].

In terms of root morphology, significant differences were found among graft combinations and the interaction between N supply and graft combination was also highly significant (*p* < 0.001) (Table 4). Compared to non-grafted control plant (Crimson Sweet), the graft combination 70-07/C.S. and 07-45/C.S. showed the highest root dry matter, total root length and root volume at both low and high N rates. On the other hand, the graft combinations 35-10/C.S. and 45-07/C.S. exhibited slightly higher root dry matter, root length and volume than non-grafted control plants, but the production was significantly lower than the graft combinations of 70-07/C.S. and 07-45/C.S. at both low and high N conditions. All these results clearly confirmed our starting hypothesis which proposes that a low yielding ‘N-inefficient’ watermelon cultivar (Crimson Sweet) can be improved when it is grafted onto ‘N-efficient’ bottle gourd rootstocks (70-07 and 07-45). The grafted N-efficient gourd genotypes 07-45 and 70-07 significantly contributed to growth and biomass production of the N-inefficient watermelon plants as compared to non-grafted control plants and thus showed a higher rootstock potential for watermelon. 

Furthermore, these results also confirmed that the selection and characterization of bottle gourd genotypes (N-efficient/N-responsive: 70-07 and 07-45, N-inefficient/N-irresponsive: 35-10 and 45-07) based on the results of *Exp. 1*, have been done correctly. 

## 4. Conclusions

Sustainability in food and agricultural production depends on the efficient use of natural resources and requires reduced N application rates in future. In this study, two screening experiments were conducted in nutrient solution growth system under 2 N doses (0.3 mM and 3.0 mM N). In the first experiment (*Exp. 1*) significant genotypic variation in N efficiency was found between the 10 local Turkish bottle gourd genotypes and two commercial watermelon cultivars. Regarding to N efficiency characteristics, four gourd genotypes (N-efficient: 70-07 and 07-45, N-inefficient: 35-10 and 45-07) were selected and used as rootstock for grafting with N-inefficient watermelon cultivar (Crimson Sweet) under 2 N doses in the second experiment (*Exp. 2*). The grafted N-efficient gourd genotypes (07-45 and 70-07) significantly contributed to growth and biomass production of the N-inefficient watermelon plants as compared to non-grafted control plants and thus showed a higher rootstock potential for watermelon. The N-efficiency of some gourd genotypes were associated with vigor root growth and active root system particularly at low N condition. These traits could be useful characters for the selection and breeding of ‘N-efficient’ bottle gourd rootstocks for sustainable agriculture in the future.

## Figures and Tables

**Table 1:** Shoot dry matter, nitrogen uptake, nitrogen concentration and total leaf area of ten bottle gourd genotypes and two watermelon cultivars grown under low (0.3 mM) and high (3.0 mM) N supply in *Expt. 1*.

*Expt. 1*	Shoot Dry Matter (g plant^−1^)		Nitrogen Uptake (mg plant^−1^)		Nitrogen Concentration (mg g dw.^−1^)		Leaf Area (cm^2^ plant^−1^)	
Genotype	Low N	High N	Low N	High N	Low N	High N	Low N	High N
01-18	7.4 de	34.8 D	104.6 e	937.8 E	14.2 hi	26.9 EF	0.14 ef	0.75F
07-45	14.6 a	41.9 A	202.3 b	1032.6 D	13.9 I	24.6 HI	0.20 ab	0.97B
35-10	7.3 de	30.2 E	106.6 e	866.5 F	14.5 gh	28.7 BC	0.15 de	0.62G
41-01	9.3 bc	37.7 C	155.5 c	1144.5 B	16.7 d	29.3 BC	0.18 c	0.93BC
42-11	10.0 b	42.8 A	146.4 cd	1089.5 C	14.6 gh	25.5 GH	0.19 bc	0.91CD
45-04	8.7 c	39.1 B	140.2 d	1157.3 B	16.2 e	29.6 B	0.18 c	0.96B
45-07	6.9 de	26.8 F	102.5 e	790.2 G	14.8 g	29.5 B	0.13 g	0.56H
47-04	9.4 bc	38.6 BC	134.8 d	932.6 E	14.4 gh	24.2 I	0.18 c	0.87D
70-07	14.7 a	43.2 A	229.2 a	1226.4 A	15.5 f	28.4 CD	0.21 a	1.26A
TR-50	7.6 d	35.1 D	139.6 d	919.8 E	18.4 c	26.2 FG	0.16 d	0.80E
C. Sweet	5.8 f	8.1 H	112.4 e	222.8 I	19.3 b	27.5 DE	0.10 h	0.12J
S. Baby	6.8 e	12.7 G	139.2 d	424.9 H	20.6 a	33.6 A	0.13 fg	0.18I
Genotype	***		***		***		***	
N rate	***		***		***		***	
Gen. × N	***		***		**		***	

Values denoted by different letters (lower- and upper-case letters for low and high N supply, respectively) are significantly different between genotypes within columns at *p* < 0.05. ns, non-significant. * *p* < 0.05, ** *p* < 0.01 and *** *p* < 0.001.

**Table 2:** Root dry matter, root:shoot ratio, root length and root volume of the ten bottle gourd genotypes and two watermelon cultivars grown under low (0.3 mM) and high (3.0 mM) N supply in *Expt. 1*.

*Expt. 1*	Root Dry Matter (g plant^−1^)		Root:Shoot (g g^−1^)		Root Length (m plant^−1^)		Root Volume (cm^3^ plant^−1^)	
Genotype	Low N	High N	Low N	High N	Low N	High N	Low N	High N
01-18	2.60 f	2.11 F	0.35 d	0.06 G	410.4 g	336.4 I	54.2 f	38.9 G
07-45	7.53 b	5.83 A	0.52 b	0.14 A	650.4 b	523.6 B	129.2 ab	94.7 AB
35-10	3.09 e	2.64 E	0.42 c	0.09 E	484.5 f	408.7 H	102.9 d	73.5 E
41-01	5.26 d	4.90 B	0.56 b	0.13 B	578.2 cd	512.4 C	113.5 cd	90.2 BC
42-11	5.52 c	4.13 D	0.55 b	0.10 D	582.6 cd	457.6 F	117.5 bc	82.8 CD
45-04	5.57 c	4.62 C	0.64 a	0.12 C	597.4 c	486.7 E	126.8 bc	91.8 AB
45-07	2.44 f	1.86 G	0.35 d	0.07 F	386.2 h	279.7 J	84.1 e	54.9 F
47-04	5.28 d	4.12 D	0.56 b	0.11 C	568.7 de	448.1 G	120.8 bc	81.1 DE
70-07	7.90 a	6.03 A	0.54 b	0.14 A	730.0 a	550.7 A	143.3 a	99.6 A
TR-50	5.11 d	4.78 BC	0.67 a	0.14 A	556.0 e	497.7 D	113.6 cd	87.5 CD
C. Sweet	1.26 h	0.56 I	0.22 e	0.07 F	229.3 j	114.5 L	26.0 g	11.7 H
S. Baby	1.78 g	0.92 H	0.26 e	0.07 F	330.2 i	205.6 K	35.5 g	19.8 H
Genotype	***		***		***		***	
N rate	***		***		***		***	
Geno. × N	***		***		**		***	

Values denoted by different letters (lower- and upper-case letters for low and high N supply, respectively) are significantly different between genotypes within columns at *p* < 0.05. ns, non-significant. * *p* < 0.05, ** *p* < 0.01 and *** *p* < 0.001.

**Table 3:** Shoot dry matter, nitrogen uptake, nitrogen concentration and total leaf area of non-grafted and grafted watermelon cultivar (Crimson Sweet) onto four bottle gourd genotypes grown under low (0.3 mM) and high (3.0 mM) N supply in *Expt. 2*.

*Expt. 2*	Shoot Dry Matter (g plant^−1^)		Nitrogen Uptake (mg plant^−1^)		Nitrogen Concent. (mg g dw^−1^)		Leaf Area (cm^2^ plant^−1^)	
Graft.Com.	Low N	High N	Low N	High N	Low N	High N	Low N	High N
C. Sweet	4.7 e	9.8 E	77.7 e	331.6 E	16.4 c	33.8 B	0.11 d	0.15 E
C.S./07-45	11.2 b	33.0 B	186.2 b	1153.6 B	16.7 bc	34.9 A	0.17 b	0.52 B
C.S./35-10	8.8 c	20.3 C	151.1 c	613.3 C	17.2 a	30.2 D	0.13 c	0.37 C
C.S./45-07	8.5 d	17.5 D	143.5 d	487.4 D	16.9 b	27.8 E	0.13 c	0.30 D
C.S./70-07	11.6 a	38.5 A	191.2 a	1266.8A	16.4 c	32.9 C	0.20 a	0.64 A
Grafting	***		***		***		***	
N rate	***		***		***		***	
Graft. × N	***		***		**		***	

Values denoted by different letters (lower- and upper-case letters for low and high N supply, respectively) are significantly different between genotypes within columns at *p* < 0.05. ns, non-significant. * *p* < 0.05, ** *p* < 0.01 and *** *p* < 0.001.

**Table 4:** Root dry matter, root:shoot ratio, root length and root volume of non-grafted and grafted watermelon cultivar (Crimson Sweet) onto four bottle gourd genotypes grown under low (0.3 mM) and high (3.0 mM) N supply in *Expt. 2*.

*Expt. 2*	Root Dry Matter (g plant^−1^)		Root:Shoot (mg plant^−1^)		Root Length (m plant^−1^)		Root Volume (cm^3^ plant^−1^)	
Graft.Com.	Low N	High N	Low N	High N	Low N	High N	Low N	High N
C. Sweet	0.94 d	0.71 E	0.20 d	0.07 B	172.7 e	144.1 E	19.6 d	14.7 D
C.S./07-45	3.14 b	2.42 B	0.28 b	0.07 B	263.5 b	218.8 B	63.0 a	44.8 A
C.S./35-10	2.30 c	2.16 C	0.26 c	0.11 A	212.2 c	202.3 C	42.5 c	37.5 B
C.S./45-07	2.25 c	1.81 D	0.26 c	0.10 A	206.9 d	174.7D	39.6 c	29.7 C
C.S./70-07	3.52 a	2.52 A	0.30 a	0.07 B	286.5 a	228.4A	55.4 b	45.6 A
Grafting	***		***		***		***	
N rate	***		***		***		***	
Graft. × N	***		***		**		***	

Values denoted by different letters (lower- and upper-case letters for low and high N supply, respectively) are significantly different between genotypes within columns at *p* < 0.05. ns, non-significant. * *p* < 0.05, ** *p* < 0.01 and *** *p* < 0.001.
